# Waiting for transgender care and its effects on health and equality: a mixed-methods population study in the Netherlands

**DOI:** 10.1016/j.eclinm.2024.102657

**Published:** 2024-05-31

**Authors:** Tim C. van de Grift, Casper Martens, Lisa van Ginneken, Margriet G. Mullender

**Affiliations:** a Department of Plastic, Reconstructive and Hand Surgery, Amsterdam UMC, Location VUmc, Amsterdam, the Netherlands; b Department of Psychiatry, Zaans Medisch Centrum, Amsterdam, the Netherlands; c Amsterdam Public Health Institute, Amsterdam, the Netherlands; d Department of Medical Psychology, Amsterdam UMC, Locations VUmc and AMC, Amsterdam, the Netherlands; e Transvisie Transgender Patient Support Group, Utrecht, the Netherlands; f Scientific Institute for Quality of Healthcare (IQ Healthcare), Radboud University Medical Center, Nijmegen, the Netherlands

**Keywords:** Transgender, Gender-affirming care, Access to care, Health promotion

## Abstract

**Background:**

Access to gender-affirming medical care has life-saving effects on transgender and gender diverse (TGD) individuals. An increasing number of TGD individuals seek care which led to waiting times of years. We assessed the duration and effects of excessive waiting for gender-affirming care.

**Methods:**

In this mixed-methods cross-sectional population study, conducted in the Netherlands between June and August 2019, all TGD individuals with experiences with receiving/applying for gender-affirming medical care could participate. Data of participants waiting for care was primarily analyzed. Demographic characteristics and requested care (multiple-choice questions) and the effects of waiting on health were surveyed (open-ended questions). Descriptive statistics were calculated per treatment, and differences in participant characteristics between those waiting for and already in care were assessed through Chi squared tests and post-hoc testing of residuals. Open text answers were thematically analyzed.

**Findings:**

Of all 975 participants, 431 (44.2%) waited for gender-affirming medical care. For 10 out of 20 treatment modalities, the median waiting period was more than a year. Participants already in care were on average higher educated and less-frequently non-binary. Subjective effects of waiting were (very) negative for most treatments. Qualitative findings showed that long waiting was associated with a range of psychosocial distress, poorer health, increasing healthcare consumption, and increased inequality between TGD individuals.

**Interpretation:**

Findings confirm that TGD individuals encounter long waiting times in multiple stages of their gender-affirming medical care. Waiting affects both physical and psychosocial health. Individual resilience and resources can mitigate these experiences, which increase inequality within this already marginalized group.

**Funding:**

Ministry of Health, Welfare and Sport of the Netherlands and 10.13039/501100023976Zorgverzekeraars Nederland (Healthcare Insurers Netherlands).

**Research in context**

**Evidence before this study**

Prior to this study, and before drafting the manuscript, Pubmed was searched using MeSH terms and title/abstract including terms on both “transgender” OR “gender diverse” OR “gender dysphoria” (and equivalents) AND terms relating to “barriers to care” OR “waiting lists” (and equivalents). To our knowledge, no studies of significant size have been published on TGD individuals on waiting lists and their experiences. Hypotheses were therefore derived from other populations, clinical practice and non-scientific sources.

**Added value of this study**

This study describes a sample of the TGD population waiting for gender-affirming care. While being a frequently-debated topic, little research data on this group has been published. This study provides an overview of the demographic characteristics of TGD individuals waiting for care, future requests for care, and the implications of extensive waiting lists. It not only quantifies these effects per type of care modality; it also provides insights into how these waiting experiences affect different domains of life, how it can result in worsening health and more extensive healthcare needs, and how it increases inequalities within the TGD population. This also exhibits individual coping strategies to mitigate the effects of waiting.

**Implications of all the available evidence**

Decreased access to gender-affirming care has a range of negative effects on TGD individuals. Qualitative data showed how waiting can result in increased healthcare consumption due to worsened (mental) health, unwanted pubertal developments, poor lifestyle and self-medicating. Also, inequality within TGD individuals likely increases with more resourceful individuals having access to foreign or informal care, and being able to cope more resiliently.

## Introduction

Gender-affirming care, including psychological, hormonal and surgical interventions, are regarded as key to the improvement of quality of life of transgender and gender diverse (TGD) individuals.^1^ TGD individuals suffering from body dissatisfaction, gender dysphoria and/or mental health problems generally benefit strongly from medical interventions that align bodily characteristics with one’s experienced gender identity. Given the repeated findings that gender-affirming care improves the health^2^, ^3^, ^4^ and a range of quality of life domains,^5^^,^^6^ multiple (non-)governmental bodies, both in the European Union^7^ and the United States,^8^ state that sufficient access to this care is or should be an individual right.

In practice, access to safe and good-quality care, including in Western countries, is far from secure for many TGD individuals. Online survey data showed that 71% of TGD individuals had stigmatizing healthcare experiences, including lack of sufficient knowledge of healthcare providers, and unwelcoming or discriminating environments.^9^ Barriers to accessing gender-affirming care specifically include issues with obtaining referrals, extensive travel time and costs, issues with sufficient reimbursement, and fear of rejection when treatment requests might not match the dominant gender binary protocols.^10^ While some barriers might be generalizable, others are likely dependent on local healthcare standards and organization of care.^11^^,^^12^ Countries with decentralized care systems have seen an increase in newly established providers, which may result in, among other issues, unclear quality and reimbursement procedures for patients. On the other hand, countries with regulated centralized reimbursed care generally have not been able to scale their facilities to the rising demand and train enough providers to ensure timely access to care. In many countries, the *de facto* waiting period to access gender-affirming care exceeds the maximal duration as dictated by healthcare laws and policies. Inability to timely access gender-affirming care may lead to psychological distress,^13^ and health risks, e.g., resulting from self-medicating sex hormones.^14^

Given the importance of sufficient access to gender-affirming care, and the range of possible consequences of prolonged suffering from gender dysphoria, the aim of this study was to assess gender-affirming care waiting experiences in a country with a centralized reimbursed healthcare system (the Netherlands). While most studies report on characteristics of TGD individuals who have already entered (or have gone through) care, this study describes those who are still waiting. With that regard, we aim to describe what types of care participants are waiting for, how long they have been/will be waiting, and what the effects of waiting are on several aspects of their lives.

## Methods

### Procedure

The present study was part of a National Transgender Survey assessing the experiences, needs and barriers experienced by TGD individuals regarding gender-affirming care in the Netherlands. Gender-affirming medical care in the Netherlands is provided in a limited number of secondary and tertiary, mostly multidisciplinary, hospital teams after referral by a general practitioner. Care is conduced according to locally adapted international guidelines in which psycho-diagnostic contacts/counseling are followed by medical care. The treatment order is largely based on patient preferences, all non-cosmetic surgeries are reimbursed, and there is no sterilization requirement to change legal documents. Participants self-selected based on their TGD identification and were eligible when residing in the Netherlands, regardless of their stage and type of healthcare. The study was initiated and conducted in a consortium of a (research) consultancy, TGD patient support organization Transvisie, and scientists from two academic hospitals: Amsterdam UMC, location VUmc (Amsterdam, the Netherlands) and Radboudumc (Nijmegen, the Netherlands). The study had a mixed-methods quantitative and qualitative cross-sectional design, using online questionnaires. Data analyses was performed sequentially: quantitative analyses of the effects of waiting times were followed by explanatory qualitative data analysis that sought to describe possible mechanisms accounting for the quantitative findings.

Participants either filled out the survey for themselves or were asked to do so for/with a minor, if their child was aged under 16 years. Recruitment took place between June and August 2019 and surveyed experiences in 2017, 2018 and 2019. Since 2019, the waiting times have been similar or have increased for most gender-affirming treatments. Given the fact the project targeted participants that were not yet enrolled in care, recruitment took place via the websites and social media channels of TGD patient support groups, LGBT/transgender interest groups and mainstream media channels. Potential participants were invited knowing the study surveyed experiences and needs regarding (accessing) gender-affirming care in the Netherlands. If interested, participants were redirected to the online questionnaire where they could provide informed consent and/or find contact details in case of questions. The survey included up to 67 questions (based on experiences with care) and took a median of 20 min to complete (IQR = 11–35: questionnaire available as Appendix). Participants received no compensation for participation.

### Ethics statement

All participants provided written informed consent for their participation. The study received ethical approval from the Amsterdam UMC, location VUmc IRB (FWA00017598).

### Participants

Due to the recruitment strategy, no estimate of the number of eligible participants reached could be given. In the Netherlands there are an estimated total of 34 thousand TGD individuals who underwent or have a wish for gender-affirming care,^15^ of which a selection has recently interacted with gender-affirming care and may also have been exposed to study recruitment communication. At study closure, 1237 TGD individuals consented and participated, of whom 975 (79%) filled out the minimally required information (i.e., background information and data on the study primary outcomes).

### Quantitative measures

The questionnaire was designed by the study consortium for the specific study purposes and received community feedback prior to participant recruitment. The final questionnaire consisted of a section on demographic and clinical background questions and an outcome section assessing waiting times and their effects. Only the measures relevant to the present study aim will be described here (survey questions are provided as Appendix). General demographic questions included multiple-choice questions on sex assigned at birth, gender identity at present (male/masculine, female/feminine, other/non-binary), educational level and ethnic background (of self and parents). Clinical questions included the aim of one’s gender transition (social, physical/medical, both or uncertain), the current status of receiving gender-affirming care (no wish, not applied yet, waiting for initial intake, in/finished care), and what further treatments were planned.

With regard to the waiting lists, participants were provided with a list of 20 gender-affirming treatment modalities available in the Netherlands (Table 1) and were asked to confirm if they were currently waiting for one or more of those treatments. When confirmed, participants received three more questions on waiting for each of these treatments (1) a multiple-choice question on the total time they had to wait as communicated by the healthcare provider (<1 month, 1–2 months, 3–6 months, 0.5–1 year, 1–1.5 year, >1.5 year, unknown), (2) a multiple-choice question asking *To which degree do you suffer from the waiting period to receive this care?* (no negative effect, little negative effect, somewhat negative effect, negative effect, very negative effect), and (3) an open question on the effects of waiting on their life (see below).

**Table 1:** Treatment modalities, waiting (duration) and effects.

Treatment modality	Number of participants waiting (%)	Median duration of waiting (Q1–Q3)		Experienced effects of waiting (%)		
				No/little negative effect	Somewhat negative effect	(Very) negative effect
Psycho-diagnostic consults^a^	188 (21.4)	More than 1.5 years	(1 to more than 1.5 years–more than 1.5 years)	6 (3.5)	30 (17.4)	136 (79.1)
Cross-sex hormone treatment	157 (17.9)	More than 1.5 years	(0.5–1 year–more than 1.5 years)	5 (4.2)	24 (20.3)	89 (75.4)
Puberty suppression	78 (8.9)	More than 1.5 years	(1 to more than 1.5 years–more than 1.5 years)	6 (10.7)	10 (17.9)	40 (71.4)
Masculinizing genital surgery	24 (4.0)	1 to more than 1.5 years	(0.5–1 year–more than 1.5 years)	3 (17.6)	5 (29.4)	9 (52.9)
Adam’s apple correction	12 (3.0)	1 to more than 1.5 years	(0.5–1 year–more than 1.5 years)	2 (20.0)	1 (10.0)	7 (70.0)
Support in coming-out	31 (3.5)	1–1.5 years	(3–6 months–1 to more than 1.5 years)	–	3 (15.0)	17 (85.0)
Psychological care or counseling	122 (13.9)	1–1.5 years	(3–6 months–more than 1.5 years)	4 (4.1)	18 (18.6)	75 (77.3)
Fertility treatments	21 (2.4)	1–1.5 years	(1–2 months–more than 1.5 years)	2 (20.0)	4 (40.0)	4 (40.0)
Hair removal therapy	18 (1.8)	1–1.5 years	(3–6 months–more than 1.5 years)	2 (13.3)	1 (6.7)	12 (80.0)
Social work	12 (1.4)	1–1.5 years	(3–6 months–1 to more than 1.5 years)	–	3 (75.0)	1 (25.0)
Facial feminization surgery	25 (2.8)	0.5–1 year	(3–6 months–more than 1.5 years)	3 (18.8)	4 (25.0)	9 (56.3)
Mastectomy	110 (19.3)	0.5–1 year	(0.5–1 year–more than 1.5 years)	1 (1.1)	11 (12.2)	78 (86.7)
Feminizing genital surgery	45 (10.2)	0.5–1 year	(3–6 months–more than 1.5 years)	2 (5.4)	9 (24.3)	26 (70.3)
Gynecological surgery	30 (5.0)	0.5–1 year	(3–6 months–1 to more than 1.5 years)	3 (17.6)	8 (47.1)	6 (35.3)
Sexual care or counseling	30 (3.4)	0.5–1 year	(3–6 months–1 to more than 1.5 years)	3 (15.8)	5 (26.3)	11 (57.9)
Vocal cord surgery	8 (2.0)	0.5–1 year	n/a	1 (25.0)	–	3 (75.0)
Speech and language therapy	10 (1.3)	0.5–1 year	(less than 1 month—more than 1.5 years)	1 (20.0)	2 (40.0)	2 (40.0)
Breast enlargement surgery	12 (2.7)	3–6 months	(1–2 months–6 to 12 months)	1 (12.5)	2 (25.0)	5 (62.5)
Physical therapy	14 (1.6)	3–6 months	(1–2 months–6 to 12 months)	3 (42.9)	2 (28.6)	2 (28.6)
Hair transplant surgery	5 (1.3)	–	n/a	1 (50.0)	–	1 (50.0)

Percentages calculated of the eligible participants.

a Includes initial intake to access medical care, n/a = not applicable.

### Qualitative measures

For each treatment participants were currently waiting for, respondents were asked the following open question: *Which consequences does this waiting period have for you personally?* Participants received unlimited space to answer each time they got this question.

### Statistical analysis

The study aimed to include a representative number of TGD individuals in the Netherlands in each stage of gender-affirming care. Given the variability in assigned sex, gender and age, as well as the large number of treatment options, the study aimed to include at least 30 participants waiting per main treatment modality (i.e., psycho-diagnostic consults, psychological care or counseling, cross-sex hormone treatment, puberty suppression treatment, mastectomy and feminizing genital surgery). After 2 months, an intermediary analysis was performed to assess sub-sample sizes, after which additional recruitment of (parents of) transgender youth was performed due to underrepresentation of this group.

Given the exploratory nature of the study, merely descriptive statistics were performed, including frequencies (%) and medians (for duration of waiting time). Exploratory analyses showed smaller sample sizes for certain treatments after which the response categories on the effects of waiting for care were combined to three (Table 1). Differences in background demographics were tested for the different groups that either did not require/apply for care, were waiting for care or were receiving/finished care, using Chi squared tests, Cramer’s V effect sizes and posthoc testing using standardized residuals. Multivariate analysis on background differences per group waiting/in care (dependent variable) was performed through multinominal logistic regression (independent variables: gender identity, sex-assigned at birth, age, education, ethnicity and transition aim). All quantitative statistical analyses were performed in SPSS statistics (version 28).

Qualitative analysis followed quantitative analysis and aimed to build on the quantitative findings on waiting times and their effects. Open text answers were analyzed thematically for all treatment modalities together to reduce analytical complexity. Data was grouped in excel files and coded by two authors (a TGD-identifying researcher and a cisgender-identifying clinician-researcher) until agreement was achieved, after which the themes were confirmed by the other authors (a TGD and a cisgender-identifying researcher). Themes were developed inductively using a phenomenological approach without prior theoretical framework or assumption.^16^ After primary themes were defined, participants quotations were selected to illustrate the findings. In defining the themes and quote selection, the authors aimed to capture as much of the range of participant experiences as possible. Data analysis was completed after reaching consensus between all authors. The study design did not allow for participant checking. Quantitative and qualitative results were presented sequentially and mixed-method data was integrated in the discussion.

### Role of the funding source

The study was funded by the Ministry of Health, Welfare and Sport of the Netherlands and Zorgverzekeraars Nederland (Healthcare Insurers Netherlands). The funders of the study had no role in study design, data collection, data analysis, data interpretation, or writing of the manuscript. T.v.d.G., L.v.G. and C.M. had access to the dataset, whereas all authors were responsible for the decision to submit for publication.

## Results

Amongst the 975 study participants, 490 identified as trans masculine (50.3%), 207 as trans feminine (31.5%) and 178 as non-binary/other (18.3%), altogether with a median age of 22 years (IQR = 17–35). For 33 (3.4%) no transition was planned, 108 (11.1%) were exploring, 268 (27.5%) were at the early stages of transition, 338 (34.7%) in the middle of transition, 97 (9.9%) were nearly finished, whereas 131 had already finished their transition (13.5%).

### Waiting for care

When combining answers for all treatment modalities, 431 transgender individuals (44.2%) were on any waiting lists at time of participation. When excluding participants without (medical) treatments planned and those with finished transitions, 53.1% of participants was waiting for care. Treatments with the largest number and/or percentage of individuals waiting, included psycho-diagnostic consults (i.e., initial intake), mastectomy, cross-sex hormone treatment, psychological care and counseling, feminizing genital surgery and puberty suppression treatment (Table 1). For ten out of the 20 treatment modalities, the median waiting list was at least one year, with a median waiting period of more than 1.5 years for initial intake, cross-sex hormone treatment and puberty suppression. The experienced effects of waiting for gender-affirming care were generally reported to be (very) negative, with somewhat more neutral answers for gynecological and masculinizing genital surgeries. Effects of waiting for care were not only associated with the median waiting time, but also with the type of intervention: possibly related to how urgent the treatment was to participants. Further exploration of these negative effects was sought in the qualitative data.

### Characteristics of individuals waiting for vs. in care

Background characteristics are displayed in Table 2 for participants with different treatment stages and requests. Participants who experienced no necessity for medical care were more frequently male-assigned at birth (P = 0.002) and identified more frequently as non-binary (P < 0.001) than the other groups. Also, this group was on average older (P < 0.001), and intended to go through social transition only, or was unsure about medical transition (P < 0.001). Similarly, participants with a treatment wish, but who had not applied yet, more frequently identified as non-binary and were more often uncertain about their transition aim (P < 0.001). Participants who were currently waiting for initial intake were on average lower educated, and more frequently intended to go through social transition only or were uncertain, as compared to those who were already in treatment or finished treatments (P < 0.001). Participants who were already in care less frequently identified as non-binary, and were somewhat higher educated, as compared to those who were not (yet) in care (both P < 0.001). The largest effect sizes of group differences were observed for gender identity and transition aim (both moderate effect sizes: Cramer’s V = 0.28 and 0.25). These factors remained statistically significant in multivariate analysis (both P < 0.001).

**Table 2:** Characteristics of participants waiting for initial intake vs. in/finished gender-affirming care, n (%).

	Total n = 852	Not required n = 26	Not applied yet n = 68	Waiting for initial intake n = 188	Participants in/finished care since 2017 n = 402	Participants in/finished care before 2017 n = 168	Test statistics
Gender							χ2 (8) = 130.4, P < 0.001
Male/masculine	427 (50.1)	4 (15.4)^a^	17 (25.0)^a^	99 (52.7)	224 (55.7)	83 (49.4)	Cramer’s V = 0.28
Female/feminine	275 (32.3)	7 (26.9)	12 (17.6)^a^	53 (28.2)	132 (32.8)	71 (42.3)^a^	
Other/non-binary	150 (17.6)	15 (57.7)^a^	39 (57.4)^a^	36 (19.1)	46 (11.4)^a^	14 (8.3)^a^	
Sex assigned at birth							χ2 (4) = 16.9, P = 0.002
Male	328 (38.5)	18 (69.2)^a^	25 (36.8)	67 (35.6)	141 (35.1)	77 (45.8)	Cramer’s V = 0.14
Female	524 (61.5)	8 (30.8)^a^	43 (63.2)	121 (64.4)	261 (64.9)	91 (54.2)	
Age, M (SD)	27.6 (15.2)	38.8 (21.0)^a^	25.3 (15.1)	21.2 (11.7)	26.9 (13.8)	35.5 (16.6)^a^	F (4,847) = 26.6, P < 0.001
Education level							χ2 (12) = 44.5, P < 0.001
University	250 (30.9)	9 (34.6)	17 (29.8)	43 (24.7)	109 (28.1)	72 (44.2)^a^	Cramer’s V = 0.14
Higher secondary	339 (42.0)	10 (38.5)	25 (43.9)	66 (37.9)	180 (46.4)	58 (35.6)	
Lower secondary	82 (10.1)	5 (19.2)	3 (5.3)	15 (8.6)	40 (10.3)	19 (11.7)	
Primary	137 (17.0)	2 (7.7)	12 (21.1)	50 (28.7)^a^	59 (15.2)	14 (8.6)^a^	
Transition aim							χ2 (12) = 150.9, P < 0.001
Social	43 (5.2)	7 (35.0)^a^	4 (6.3)	20 (11.0)^a^	10 (2.6)^a^	2 (1.2)^a^	Cramer’s V = 0.25
Medical	77 (9.3)	3 (15.0)	5 (7.8)	17 (9.3)	41 (10.5)	11 (6.6)	
Both	658 (79.9)	6 (30.0)^a^	40 (62.5)	124 (68.1)	337 (86.0)	151 (91.0)	
Uncertain	46 (5.6)	4 (20.0)^a^	15 (23.4)^a^	21 (11.5)^a^	4 (1.0)^a^	2 (1.2)^a^	
Ethnic background							P > 0.05
Netherlands	823 (96.7)	23 (88.5)	65 (95.6)	179 (95.2)	390 (97.3)	166 (98.8)	Cramer’s V = 0.09
other Western	16 (1.9)	1 (3.8)	2 (2.9)	5 (2.7)	7 (1.7)	1 (0.6)	
non-Western	12 (1.4)	2 (7.7)^a^	1 (1.5)	4 (2.1)	4 (1.0)	1 (0.6)	

a Difference in posthoc testing.

In addition, participants also described latent treatment wishes (latent for future providers/policy makers): treatment modalities with highest number of people wanting it, but had not applied yet included mastectomy (n = 209, 36.7%), gynecological surgery (n = 189, 31.6%), cross-sex hormone therapy (n = 182, 20.7%), masculinizing genital surgery (n = 141, 23.6%), speech and language therapy (n = 123, 15.5%), feminizing genital surgery (n = 112, 25.3%), and facial feminization surgery (n = 100, 11.4%).

### Qualitative findings

A total of 339 participants provided qualitative data on the effects of waiting for gender-affirming care. Open text analysis revealed three negative and one positive theme.

### The wide range of psychosocial domains affected by waiting

Waiting for gender-affirming care resulted in a range of psychosocial problems with significant collective suffering. In many, the waiting affected most areas of life, which could lead to a complete standstill and/or experiences of loss. Participants reported increased or prolonged gender-related distress (e.g., gender dysphoria, body dissatisfaction and disgust), problems with oneself (e.g., confusion about self, suppressing oneself, low self-esteem, and feeling unauthentic), emotional problems (e.g., stress, restlessness, frustration, worries, feeling unimportant, a lack of self-determination or perspective, loneliness, hopelessness and shame).

“I feel a lot of confusion, insecurity and discomfort with my body, how other people see me, and the need to talk with a professional about this. The current situation makes me very insecure.” Non-binary, 29 years

With regard to the social problems people experienced due to waiting, people reported they were frustrated that their transition process stagnated and they were forced to stay “in the closet” longer. Many felt their lives were on hold, and they tended to socially withdraw, have poorer school/work functioning, and defer initiating new contacts, jobs, study etc. until medical transition would start. Due to deferral of medical treatments, participants reported continuation of problems in social encounters, such as being misgendered, unwanted social self-disclosure, not being accepted by others without proper diagnosis/care, or even physical aggression. Lastly, participants also reported how the extensive waiting led to a negative view on healthcare, including feeling distrust towards healthcare professionals and feeling unseen and unimportant. As a 26-years old transmasculine participant mentioned, *“I feel like I’m not important. They ‘throw’ you on a waiting list, won’t give you any contact person to discuss your situation and tell you to wait for another 2 years”*. When seeking alternative options, people often experienced problems finding other providers and therefore had no one to turn to with their problems.

“I feel like I cannot continue with my life. Everyone I meet gets to know me as someone who ‘wants to become a man’ and not as someone who ís a man. I haven’t swum for years because I just do not want to with this body. I just cannot be fully myself and at the same time be comfortable with how I look.” Transmasculine, 20 years.

“It makes me depressed and I feel like I have to postpone my life as I do not feel at home in myself. It makes me very insecure about my future, as well as about contact with others. Sometimes I tend to isolate myself as I become so distressed and do not want to be confronted with other people’s image of me. It has a very negative impact on my school performance and my ability to concentrate. I ruminate a lot.” Transmasculine, 18 years.

### Poorer health status at onset of care and more extensive treatments due to extensive waiting

In line with the aforementioned, participants described how the long waiting period, especially for initial intake and psychological counseling, was detrimental to their physical and mental health status. Feelings of hopelessness and lack of perspective resulted in developing additional mental health conditions such as clinical anxiety and depression, self-harm and suicidality. Participants described how they felt these mental health illnesses required additional treatments, that they were avoidable, and that they feared it would complicate the progress of their intake. Participants feared they would require additional mental health screenings and/or that the medical transition may have less positive effects on their well-being once they could start.

Two types of physical problems arose in relation to the waiting period. Due to gender dysphoric feelings, multiple participants described how they were not able to have the healthy lifestyle they wished, that they experienced physical symptoms from body modifying behavior (e.g., chest binding), or how they developed eating problems or obesity. Such conditions were known to complicate eligibility for (surgical) gender-affirming treatments that require good health and body mass indexes within set margins.

“Having breasts is a big problem in my passability, with a binder I cannot cover them sufficiently. I suffer a lot from irritation, shortness of breath and movement, and pain. Therefore I cannot wear it always: therefore I have to avoid those activities or come out as trans man.” Transmasculine, 29 years

Some older participants on waiting lists feared they would be too old and become no longer eligible for any gender-affirming treatments as a result of the long waiting. A large number of (parents of) transgender minors described severe distress as a result of ongoing pubertal developments while waiting for puberty suppression treatments. Such developments not only induced mental health problems, but also produce irreversible physical changes such as voice deepening, and hair or breast growth. Many felt they would have to undergo additional surgical treatments as a result or had to cope with sub-optimal outcomes due to the extensive waiting.

“I have been waiting all my life to be myself and now I have to wait and wait and wait. I am afraid I become more masculine if I do not get the blockers in time and that makes me very nervous. I think about it all day and it distracts me from school.” Transfeminine, 14 years.

“When we visited the GP in February his [their child’s] body started to develop: now his body is ‘finished’ and fully developed into the other sex. It will be much more difficult to modify things as compared to when we would have been able to intervene earlier” (Parent of) transmasculine, 14 years.

### Increased inequality as a result of long waiting periods

Transgender individuals described different abilities to mitigate the negative effects of waiting, and to lesser extent to shorten waiting altogether. Participants also shared their strategies for resilient coping with waiting. As a result of individual differences in factors such as health literacy, financial resources, level of social support and resilience, or lack thereof, inequalities in the negative effects of waiting were apparent in the participating sample. Some (parents of) transgender individuals had healthcare professionals in their network, were able to seek care abroad or were able to access alternative care while waiting.

“Not much [on the negative effects of waiting] as we are lucky to have expertise nearby (a child and adolescent psychiatrist who had been working for the gender clinic, and a transgender man) and both parents are physician and have been able to inform themselves” (Parent of) transmasculine, 7 years.

“[…] Now others are in charge of your process, which is very frustrating. This led me to seeking care abroad.” Transmasculine, 18 years.

Individuals with sufficient resilience and support reported less interference with their lives, while others resorted in risky behavior such as self-medication or unsupported transition.

“Now that I have decided to take action for my own wellbeing, it is difficult to wait 60–70 weeks or longer even for just a first intake with experts. I would prefer not to carry out my ‘experiments’ (breast binding, changing clothes and socially presenting as male) without qualified support, but if feels like wasted time in which I could be happier than just waiting without doing anything.” Transmasculine, 36 years.

### Positive effects of waiting

In some instances, participants described positive effects from waiting for gender-affirming care. This was, however, merely described by participants that had already entered care and were waiting for surgical treatments later in their transition process. Participants described how waiting periods before extensive surgical procedures, such as genital surgeries, provided them with time to mentally prepare and to get into good physical condition to recover more effectively.

“On the other hand, it [waiting period] gives me time to work on facilitating matters such as my physical health (losing weight) and provides me with space to experiment with smaller things (e.g., clothes and makeup).” Transfeminine, 36 years.

## Discussion

Using non-clinical sampling, we were able to describe a generally understudied group of TGD individuals: those waiting for gender-affirming care. The mixed-methods approach enabled to both quantify waiting for gender-affirming care and further describe the effects of waiting. The study highlighted that waiting lists significantly exceed the standards for the maximum acceptable waiting time for healthcare in the Netherlands: i.e., 14 weeks for non-acute mental healthcare and 4–7 weeks for hospital care. Furthermore, demographic characteristics of those at waiting lists differed from those already in care, including an overrepresentation of non-binary-identifying participants in the first group. Lastly, the significant burden caused by waiting became clear from the participants’ stories, which described how extensive waiting further marginalized this group and was detrimental to their health. Some participants described how they were also able to regain some agency by developing adaptive coping strategies.

When considering only the number of TGD individuals with a wish for gender-affirming treatment, 53 percent of participants were currently on a waiting list for care. The treatments participants waited for highlighted that TGD individuals experience both barriers when first entering gender-affirming care (i.e., for psycho-diagnostic care), as well as when requesting further treatment afterwards. This included both mental health, and medical gender-affirmative care. In practice this results in “stacked” waiting lists, where TGD individuals sequentially wait for consecutive treatments in their medical transition. As a result, insufficient clinical capacity adds to the already reported barriers that TGD individuals experience when entering care, including a lack of knowledge, lack of reimbursement, insensitive healthcare staff, and normative treatment protocols.^10^^,^^11^ Healthcare systems failing to meet the care needs of TGD individuals in an equitable way may therefore perpetuate or possibly worsen the marginalized position of this group with regard to their social participation and general health.^17^^,^^18^ Similar patterns have been described for marginalized racial minority groups^19^ and people with disabilities.^20^

Furthermore, we observed a difference in characteristics of participants that were already enrolled in gender-affirming care, vs. those still waiting for initial intake or considering to apply for care. Noticeably, those already in care less-frequently identified as non-binary and were on average more educated. These findings are consistent with two hypotheses with empirical support: the changing characteristics of TGD individuals applying for care, and barriers to care certain groups of TGD individuals may experience. With regard to the first, not only a significant increase in the number of TGD individuals applying for care has been reported, multiple cohort studies from different geographical regions have also described a change in the demographic characteristics of care applicants (e.g., Zhang et al.^21^). This includes an increase in the proportion of trans masculine and non-binary-identifying people. It is known that individuals with non-binary identities may present with specific psychosocial challenges and difficulties when accessing care, which can be normative and exclusive to this group.^22^^,^^23^ Secondly, the group differences may signal barriers that less educated and non-binary individuals can experience when entering gender-affirming care, although the study design did not allow for definite conclusions on this. Lower education has been linked to less health literacy and lesser access to healthcare before.^24^ Non-binary individuals are known to experience reduced access to gender-affirming care due to factors such as, being less likely to have a GP and thus be referred,^23^ having poorer mental health and possibly being assessed as less fit to be considered for referral,^22^ and expectations of the treatment protocols as binary, leading to anticipated rejection.^10^ The qualitative data in this study did not signal significant differences in how long waiting lists are experienced in binary vs. non-binary identifying TGD individuals. Adequate knowledge on TGD subgroups that are less likely to access gender-affirming healthcare is foundational when creating equitable systems of care.

In addition to the quantitative data describing the durations of waiting and the percentage of people experiencing negative effects, participants’ open text narratives provided further understanding *how* these effects developed. Overall, participants reported the highest burden of waiting before initial intake: many described the start of their gender *journey* with a process of introspection and obtaining a referral for gender-affirming care. When, after the actual referral, being confronted with waiting lists of over 1.5 years, this often triggered hopelessness and increased dysphoria. Since data collection, in the Netherlands, as in many other Western countries, waiting times have further increased for much of gender-affirming medical care. Waiting time has especially increased for initial intake and more specialized treatments (e.g., current waiting times for the largest center of expertise in the Netherlands: more than 3 years for initial intake and another more than 2.5 years for phalloplasty). In the absence of alternative providers, people described the lack of agency and not feeling seen, as detrimental to their mental health. Agency is a known trait that can buffer between stressful life events and mental health.^25^ A lack thereof, likely contributes to worsened mental health. To subsequently cope with experiences of gender dysphoria participants described socially isolating themselves and not coming out as TGD, resulting in feelings of unauthenticity or loneliness. Such factors, including identity concealment, have been linked to internalizing mental health problems and substance use in sexual minority groups before.^26^ Participants reported developing clinical psychiatric diagnoses on top of their gender dysphoria, requiring additional care, resulting in increased healthcare consumption and non-specialized mental health providers substituting/bridging gender-affirming care. At the same time, participants reported distrust and disappointment to gender-affirming healthcare professionals due to the waiting. Frustration and delayed access to gender-affirming care have been reported before.^27^ Moreover, provider-patient trust is strongly correlated to the quality of the working relationship, patient satisfaction and overall treatment outcomes.^28^ Extensive waiting may therefore have negative effects reaching beyond the waiting period only. Long waiting was reported for all treatment modalities, but was experienced specifically distressing in case of most urgent care and when not having accessed care at all. The lack of access to puberty suppression care was reported as having (highly) negative effects due to irreversible physical changes, as well as the psychosocial stagnation in an early developmental age.

While TGD individuals experience on average more social stigma and marginalization than cisgender individuals, experiences within this group may greatly differ. On the other hand, the level of resilience may protect against stress and limit the negative effects on wellbeing.^29^ The different degrees to which participants showed resilience to waiting-related distress increased inequality within the population. Those with less adaptive problem-solving skills, and/or less social support, financial resources and other facilitating factors, reported further marginalization during the process of waiting. On the other hand, participants that did have such resources, were able to mitigate the negative effects or even sought alternative access to care (e.g., through receiving out-of-pocket paid services by foreign providers). To multiple participants, waiting was so stressful, resulting in severe depression and/or suicidality, that further deferral of prescribed hormone treatment was no option. They therefore resorted to self-medicating unregulated hormone use through the internet or informal contacts. While being aware of the possible health risks, the mental health distress was experienced as worse. Coping with waiting through self-medicating has been described in some samples before and is associated with significant health risks.^13^^,^^30^ Altogether, the extensive waiting for gender-affirming care increases marginalization and inequality within TGD population, and promotes risk-taking behavior. Policy makers should be aware of mechanisms of how substandard access to gender-affirming care relates to inequality and marginalization.

The strength of this study was the large sample size that describes characteristics and experiences of TGD individuals currently waiting for gender-affirming care. The broad non-clinical recruitment strategy enabled to reach individuals infrequently described so far. The mixed-methods approach allowed for both quantification of waiting and the effects, but also gained deeper understanding of underlying processes. This study has also several limitations. The study recruitment and participation relied on self-selection of participants. A formal clinical diagnosis and objectified waiting period was therefore not available to compare with the self-reported data. Also, given the study recruitment advertisements, participants knew the study surveyed healthcare access and experiences, which may have resulted in a bias towards individuals experiencing more negative effects of waiting. The recruitment through support groups and social media, and the lengthy digital survey, may have skewed participants possibly in TGD individuals with better psychosocial functioning, increasing non-response bias. This might be particularly the case for participating TGD minors after oversampling this group (extra recruitment efforts after intermediary analysis). Also, the study performed cross-sectional unmatched comparisons, making it impossible to draw definite causal conclusions. Lastly, given the multiple project’s aims, this study used self-constructed measures (for quantitative data) and open text answers (for qualitative data) to explore the subjects of interest. Similarly, the nature of the qualitative data collection should be seen as mostly explorative as the study design did not allow for further exploration of the experiences or performing member checks of the analyses. This leaves room for further in-depth study of topics such as mental health, resilience, access to care, and waiting lists using standardized measures such as waiting list registries, validated surveys, and/or semi-structured interviews. This also counts for the role of possible confounding in interpreting the observed associations, such as the relationship between access to care and transition aim, and the possible confounding role of psychosocial characteristics.

This study described the far-reaching consequences of the long waiting lists for gender-affirming care. It showed the highest burden before initial intake, but also highlighted the long waiting periods after entering medical care. Waiting lists are barriers to care, which is likely higher for some subgroups over others (e.g., non-binary or less educated groups), but also increases inequality within the TGD population and leads to harmful behavior such as substance use or self-medication. This probably results in increased healthcare consumption, but also decreased patient-provider trust once entering care. Clinicians, researchers and policy makers should be aware of the many effects of excessive waiting when promoting health, equitable care, and ultimately the wellbeing of TGD individuals with a wish to undergo medical transition.

## Contributors

Conceptualization: T.v.d.G., C.M. and L.v.G.; Data curation: T.v.d.G. and C.M.; Formal analysis: T.v.d.G., C.M. and M.M.; Investigation: T.v.d.G., C.M. and L.v.G.; Methodology: T.v.d.G. and M.M.; Project administration: T.v.d.G., C.M. and L.v.G.; Resources: T.v.d.G., C.M. and L.v.G.; Supervision: M.M.; Validation: T.v.d.G., C.M., L.v.G. and M.M.; Writing—original draft: T.v.d.G.; Writing–review & editing: T.v.d.G., C.M., L.v.G. and M.M.

## Data sharing statement

The data that support the findings of this study are available on request from the corresponding authors. The data are not publicly available due to privacy or ethical restrictions.

## Declaration of interests

This study was funded by funded by the Ministry of Health, Welfare and Sport of the Netherlands and Zorgverzekeraars Nederland (Healthcare Insurers Netherlands). The researchers worked independently and funders had no influence in any of the research’ stages.

## References

[1] Coleman E., Radix A.E., Bouman W.P. (2022). Standards of care for the health of transgender and gender diverse people, version 8. Int J Transgend Health.

[2] Bränström R., Pachankis J.E. (2020). Reduction in mental health treatment utilization among transgender individuals after gender-affirming surgeries: a total population study. Am J Psychiatr.

[3] Almazan A.N., Keuroghlian A.S. (2021). Association between gender-affirming surgeries and mental health outcomes. JAMA Surg.

[4] White Hughto J.M., Reisner S.L. (2016). A systematic review of the effects of hormone therapy on psychological functioning and quality of life in transgender individuals. Transgender Health.

[5] van Leerdam T.R., Zajac J.D., Cheung A.S. (2023). The effect of gender-affirming hormones on gender dysphoria, quality of life, and psychological functioning in transgender individuals: a systematic review. Transgender Health.

[6] van de Grift T.C., Elaut E., Cerwenka S.C., Cohen-Kettenis P.T., Kreukels B.P.C. (2018). Surgical satisfaction, quality of life, and their association after gender-affirming surgery: a follow-up study. J Sex Marital Ther.

[7] Europe Co (2009). Rights CfH.

[8] (2021). Equality NCfT. Know your rights in health care. https://transequality.org/know-your-rights/health-care.

[9] Kosenko K., Rintamaki L., Raney S., Maness K. (2013). Transgender patient perceptions of stigma in health care contexts. Med Care.

[10] Ross M.B., van de Grift T.C., Elaut E. (2021). Experienced barriers of care within European treatment seeking transgender individuals: a multicenter ENIGI follow-up study. Int J Transgend Heal.

[11] Puckett J.A., Cleary P., Rossman K., Mustanski B., Newcomb M.E. (2018). Barriers to gender-affirming care for transgender and gender nonconforming individuals. Sex Res Soc Policy.

[12] Koehler A., Strauss B., Briken P., Szuecs D., Nieder T.O. (2021). Centralized and decentralized delivery of transgender health care services: a systematic review and a global expert survey in 39 countries. Front Endocrinol.

[13] Linander I., Alm E. (2022). Waiting for and in gender-confirming healthcare in Sweden: an analysis of young trans people’s experiences. Eur J Soc Work.

[14] Pop M.L., Højgaard A.D., Bruun N.H., Giami A. (2022). An investigation of self-medication among gender-diverse individuals: comparison between a clinical sample and a survey sample. J Sex Med.

[15] Kuyper L. (2012). Transgenders in Nederland: prevalentie en attitudes. Tijdschr Seksuol.

[16] Sundler A.J., Lindberg E., Nilsson C., Palmer L. (2019). Qualitative thematic analysis based on descriptive phenomenology. Nurs Open.

[17] Andersen R.M., Davidson P.L., Baumeister S.E. (2007). Changing the US health care system: key issues in health services policy and management 3a edición.

[18] Gulliford M., Figueroa-Munoz J., Morgan M. (2002). What does’ access to health care’ mean?. J Health Serv Res Policy.

[19] Mayberry R.M., Mili F., Ofili E. (2000). Racial and ethnic differences in access to medical care. Med Care Res Rev.

[20] Krahn G.L., Hammond L., Turner A. (2006). A cascade of disparities: health and health care access for people with intellectual disabilities. Ment Retard Dev Disabil Res Rev.

[21] Zhang Q., Rechler W., Bradlyn A. (2021). Changes in size and demographic composition of transgender and gender non-binary population receiving care at integrated health systems. Endocr Pract.

[22] Richards C., Bouman W.P., Seal L., Barker M.J., Nieder T.O., T’Sjoen G. (2016). Non-binary or genderqueer genders. Int Rev Psychiatr.

[23] Clark B.A., Veale J.F., Townsend M., Frohard-Dourlent H., Saewyc E. (2018). Non-binary youth: access to gender-affirming primary health care. Int J Transgenderism.

[24] Van Der Heide I., Wang J., Droomers M., Spreeuwenberg P., Rademakers J., Uiters E. (2013). The relationship between health, education, and health literacy: results from the dutch adult literacy and life skills survey. J Health Commun.

[25] Thoits P.A. (2006). Personal agency in the stress process. J Health Soc Behav.

[26] Pachankis J.E., Mahon C.P., Jackson S.D., Fetzner B.K., Bränström R. (2020). Sexual orientation concealment and mental health: a conceptual and meta-analytic review. Psychol Bull.

[27] Pullen Sansfaçon A., Temple-Newhook J., Suerich-Gulick F. (2019). The experiences of gender diverse and trans children and youth considering and initiating medical interventions in Canadian gender-affirming speciality clinics. Int J Transgenderism.

[28] Calnan M., Rowe R. (2006). Researching trust relations in health care: conceptual and methodological challenges–an introduction. J Health Organisat Manag.

[29] Masten A.S., Reed M.-G.J. (2002). Handbook of positive psychology.

[30] Mepham N., Bouman W.P., Arcelus J., Hayter M., Wylie K.R. (2014). People with gender dysphoria who self-prescribe cross-sex hormones: prevalence, sources, and side effects knowledge. J Sex Med.

