# Supplementary material for: Waiting for transgender care and its effects on health and equality: a mixed-methods population study in the Netherlands
Source: eClinicalMedicine. 2024 May 31;73:102657. doi: 10.1016/j.eclinm.2024.102657 (PMC11169950; doi:10.1016/j.eclinm.2024.102657)
Supplement: Appendix [file mmc1.docx]

**Translated questionnaire (automated from Dutch)**

Introduction

You are about to complete the questionnaire "Experiences and needs in (trans)gender care." This questionnaire was developed by Transvisie and Zorgvuldig Advies. Zorgvuldig advies has been tasked by Health Insurers Netherlands and the Ministry of Health, Welfare and Sport to help alleviate the bottlenecks in (trans)gender care. This research is part of that assignment. The purpose of the questionnaire is to better understand how gender care is experienced and what needs exist. This is necessary to help caregivers, health insurers, and municipalities address issues and improve care. If you are a parent or guardian of a child aged 16 or older with experience or a need for gender care, unfortunately, this questionnaire is not intended for you. In that case, we ask you to present this questionnaire to your child. In this questionnaire, we often use the term gender care. By this, we mean all guidance and care provided to transgender individuals and individuals with questions about their gender identity. Because for the latter group it is not necessarily about transgender care, we will refer to the total care and guidance in this questionnaire as gender care from now on instead of transgender care. We ask you to take the time to complete the questionnaire; it may take over half an hour. Thank you in advance for your time and assistance. To protect your privacy, we process your answers confidentially and anonymously according to the applicable privacy guidelines. This means that they cannot be traced back to you as an individual. Data will be kept for a maximum of 2 years. By completing the questionnaire, you consent to the use of the data for the purposes described above. If you have any questions about the questionnaire or the processing of your data, please send an email to: [masked].

Background Characteristics

With the first question in this questionnaire, we ask for your age to determine whether you can complete this questionnaire yourself, together with, or by one of your parents/guardians.

V1 Which situation applies to you:

o I have personal experience with or a need for gender care and am 16 years or older (1)

o I have personal experience with or a need for gender care and am younger than 16 years (2)

o I am a parent/guardian of a child younger than 16 years with experience with or a need for gender care (3)

Display This Question:

If V1 = I have personal experience with or a need for gender care and am younger than 16 years

Due to privacy legislation, we do not approach individuals under the age of 16 for this research. However, we would like to map out the wishes, experiences, and needs of people with questions about their gender identity and transgender individuals under 16 in this research. Therefore, we kindly ask you to pass this questionnaire on to your parents so that they can complete the questionnaire (together with you) about your situation.

Display This Question:

If V1 = I am a parent/guardian of a child younger than 16 years with experience with or a need for gender care

It is important for this research to map out the wishes, experiences, and needs of people with questions about their gender identity and transgender individuals under 16 years old. However, due to privacy legislation, we do not approach them directly for this research. Therefore, we would like to ask you as a parent/guardian to complete this questionnaire with your child. The questions are asked in the "you" form, but if you complete the questionnaire as a parent/guardian without your child, please put yourself in your child's situation and try to answer the questions accordingly.

V2 What is your age? (age of your child)

________________________________________________________________

Now we would like to ask you a few background questions about you and your parents. We do this to be able to assess later to what extent the group of participants in this research corresponds to a reflection of the national population composition, as is available, for example, from CBS. These questions are completely separate from your age. Of course, these questions are also completely anonymous and are only used to characterize the total research population.

V3 What is the highest level of education you and your parent(s) or guardian(s) have completed?

University, college (1)

Havo, vwo, mbo2-4 (2)

Vmbo, mavo, mbo1 (3)

Primary education (6)

Do not know/do not want to say (7)

I (1)

Parent/guardian 1 (4)

Parent/guardian 2 (5)

V4 In which country/continent were you born? And your parents/guardians?

Netherlands (1)

Europe (but not in the Netherlands or Turkey), North America, Indonesia, Japan, Australia, New Zealand or Pacific Islands (3)

Turkey, Africa, Asia (except Indonesia and Japan), or Latin America (7)

Do not know/do not want to say (8)

I (1)

Parent/guardian 1 (4)

Parent/guardian 2 (5)

V5 What gender was registered at birth in the birth register?

o Male (1)

o Female (2)

V6 How do you identify (most strongly)?

o Male (1)

o Trans man (2)

o Female (3)

o Trans woman (4)

o Non-binary (5)

o Gender questioning (6)

o Other, namely: (7) ________________________________________________

V7 In your case - in the past, present, or future - is there a transition? If so, what stage of your transition are you in?

With transition, we mean the development that you want to undergo - regardless of which medical and/or social steps you choose in this process.

o Not applicable: no transition (1)

o I am still exploring (2)

o I am at the beginning of my transition (3)

o I am in the middle of my transition (4)

o I have almost completed my transition (7)

o I completed my transition in the past 12 months (5)

o I completed my transition over a year ago (6)

Display This Question:

If V7 != Not applicable: no transition

V8 Is it (likely) a medical or social transition in your case, or both?

A medical transition refers to the medical treatments someone receives to adjust their body to their gender identity. (e.g., puberty blockers, treatment with sex hormones and/or surgeries) A social transition is living in a role other than the one assigned at birth. This can be of the other gender or a gender role that is more detached from both genders.

o Social transition (1)

o Medical transition (2)

o Both: social and medical transition (3)

o I don't know yet (4)

V9 Have you (now or in the past) received psychological and/or medical gender care?

o Yes (7)

o No, but I have a need for it (1)

o No, and I don't need it either (5)

Skip To: V14 If V9 = No, and I don't need it either

Display This Question:

If V9 != No, and I don't need it either

And V9 != No, but I have a need for it

V12.a Now follows a question about any other limitations, conditions, or illnesses. This question is asked because certain limitations, conditions, or illnesses may affect the gender care you receive. We would like to know if this was the case for you. Do you currently or have you ever had any of the following limitations, conditions, or illnesses? (multiple answers possible)

▢ ADHD, ADD or concentration problems (1)

▢ Autism (Autism Spectrum Disorder (ASD), classic autism, Asperger's syndrome, or PDD-NOS (including the subgroup McDD)) (2)

▢ Psychological condition (e.g., depression, psychosis, bipolar disorder, schizophrenia, anxiety disorder, borderline personality disorder, eating disorder) (3)

▢ Chronic condition/illness (migraine, respiratory disease, skin condition, diabetes, chronic fatigue syndrome, rheumatism, osteoarthritis, MS, muscular dystrophy, cancer, epilepsy, cardiovascular disease, gastrointestinal disorder) (4)

If What is your age? (age of your child) Text Response Is Less Than or Equal to 20

▢ Toilet training issues (fecal and urinary problems) (9)

▢ Intellectual disability (5)

▢ An intersex condition or DSD (Difference in Sex Development) (10)

▢ Other condition/illness, namely ... (6) ________________________________________________

▢ ⊗I do not want to say (7)

▢ ⊗None of the above limitations, conditions, or illnesses (8)

Display This Question:

If V12.a , ADHD, ADD or concentration problems Is Displayed

And V12.a != I do not want to say

And V12.a != None of the above limitations, conditions, or illnesses

V12.b Did your limitation, condition, or illness play a role in or during the gender care you received?

o No, the gender care and my limitation/condition/illness were unrelated (1)

o Yes, my limitation/condition/illness played a role in gender care: my healthcare providers took it into account in a good way (2)

o Yes, my limitation/condition/illness played a role in gender care: my healthcare providers did not take it into account enough (3)

o Yes, my limitation/condition/illness played a role in gender care: my healthcare providers poorly took it into account (4)

Display This Question:

If V12.b , No, the gender care and my limitation/condition/illness were unrelated Is Displayed

V12.bT Can you explain your answer?

________________________________________________________________

________________________________________________________________

________________________________________________________________

________________________________________________________________

________________________________________________________________

V13 Have you been diagnosed with gender dysphoria and by whom? (you may select more than one answer) N.B.: the abbreviation GGZ stands for "mental health care"

▢ Yes, by a psychologist/psychiatrist at a gender center (at an academic/university medical center) (4)

▢ Yes, by a psychologist/psychiatrist at a gender-specialized mental health care institution (3)

▢ Yes, by a psychologist/psychiatrist at a general mental health care institution (7)

▢ Yes, by someone else, namely: (5) ________________________________________________

▢ ⊗No (1)

▢ ⊗I don't know (6)

V15 Below you see a number of forms of guidance or treatment, which you may currently be using or planning to use. Can you indicate for each type of care whether it applies to you and in which phase of treatment you are? N.B.: If you have received a certain form of care before 2017 and you have also received this care in or after 2017 (including any aftercare or follow-up appointments), then select the answer option "Received care in or after 2017".

Not desired/N.A. (1)

Desired, not yet enrolled (2)

On waiting list (3)

Care completed/stopped before 2017 (5)

Received care in or after 2017 (4)

I don't know (yet) (6)

(Psychological) diagnostic examination, intended to gain access to medical treatment (1)

Psychological care or guidance (2)

Assistance with coming out (3)

Social work (4)

Sexological care or guidance (5)

Puberty suppression (6)

Treatment with (sex) hormones (22)

Hair removal (e.g., facial or genital) (7)

Breast augmentation (9)

Breast removal (10)

Facial surgery (11)

Adam's apple correction (12)

Speech therapy (13)

Voice surgery/Vocal cord correction (to raise the voice) (14)

Fertility care (for questions about and treatment regarding fertility) (15)

Gynecological surgery (removal of the uterus and/or ovaries) (16)

Genital surgery (removal of vagina (colpectomy)/creation of male genital characteristics (penis, testicular implants, and/or erection prostheses)) (17)

Genital surgery (removal of penis and testes/creation of vagina (vaginoplasty)) (23)

Hair transplantation (18)

Physical therapy (e.g., pelvic floor or hand) (19)

Other care, namely: ... (20)

V16 You have indicated that this form of care is not applicable or desired for you: ${lm://Field/1}. Why not?

▢ ⊗Cannot be in my situation (1)

▢ ⊗Do not find it necessary/do not have a need for it (2)

▢ Would like to but the medical risks are too great for me (3)

▢ Would like to but cannot due to my health (5)

▢ Would like to but the care is not covered by insurance or the coverage is unclear (4)

▢ Would like to but I expect insufficient results (6)

▢ Other, namely ... (9) ________________________________________________

V17 You are on the waiting list for the following form of care: [type of care inserted]. How long is the (most recent) total waiting time that you have been told, from your registration until your turn?

oLess than a month (1)

o1 to 2 months (2)

o3 to 6 months (3)

o6 months to a year (4)

oA year to a year and a half (5)

oMore than a year and a half (7)

oI have no idea (6)

V19 How do you experience this waiting time for [type of care inserted]?

o Too short (1)

o Short (2)

o Good (3)

o Long (4)

o Too long (5)

o I don't know (yet) (6)

V20 To what extent are you bothered by the waiting time to receive this type of care: [type of care inserted]?

o Not bothered at all (1)

o Hardly bothered (2)

o Somewhat bothered (3)

o Much bothered (4)

o Very much bothered (5)

o I don't know (yet) (6)

V21 What consequences does this waiting time have for you personally?

________________________________________________________________

________________________________________________________________

________________________________________________________________
